# Implications of TGFβ Signaling and CDK Inhibition for the Treatment of Breast Cancer

**DOI:** 10.3390/cancers13215343

**Published:** 2021-10-25

**Authors:** Joseph T. Decker, Jeffrey A. Ma, Lonnie D. Shea, Jacqueline S. Jeruss

**Affiliations:** 1 Department of Biomedical Engineering, University of Michigan, Ann Arbor, MI 48109, USA; jtdecker@umich.edu (J.T.D.); jeffama@umich.edu (J.A.M.); ldshea@umich.edu (L.D.S.); 2 Department of Surgery, University of Michigan, Ann Arbor, MI 48109-5932, USA

**Keywords:** breast cancer, CDK inhibitor, TGFβ, SMAD3

## Abstract

**Simple Summary:**

Small molecules that inhibit cyclin dependent kinases (CDKs) have great potential for the treatment of breast cancer and have been implemented in the standard of care for some patients with metastatic disease. As the indications for CDK inhibitors continue to expand, and it is crucial to understand the mechanism of action of these drugs and treatment interactions with other targeted therapies. Accordingly, this review discusses subtype-specific systemic breast cancer treatment, the effects of signaling through transforming growth factor beta (TGFβ), and the unique potential for implementation of CDK inhibitor therapy.

**Abstract:**

TGFβ signaling enacts tumor-suppressive functions in normal cells through promotion of several cell regulatory actions including cell-cycle control and apoptosis. Canonical TGFβ signaling proceeds through phosphorylation of the transcription factor, SMAD3, at the C-terminus of the protein. During oncogenic progression, this tumor suppressant phosphorylation of SMAD3 can be inhibited. Overexpression of cyclins D and E, and subsequent hyperactivation of cyclin-dependent kinases 2/4 (CDKs), are often observed in breast cancer, and have been associated with poor prognosis. The noncanonical phosphorylation of SMAD3 by CDKs 2 and 4 leads to the inhibition of tumor-suppressive function of SMAD3. As a result, CDK overactivation drives oncogenic progression, and can be targeted to improve clinical outcomes. This review focuses on breast cancer, and highlights advances in the understanding of CDK-mediated noncanonical SMAD3 phosphorylation. Specifically, the role of aberrant TGFβ signaling in oncogenic progression and treatment response will be examined to illustrate the potential for therapeutic discovery in the context of cyclins/CDKs and SMAD3.

## 1. Introduction

Breast cancer is the most common cancer diagnosed among women and the second leading cause of cancer-related death among women in the United States after lung cancer [1]. Annually, there are approximately 284,000 new cases of invasive breast cancer, and 49,000 cases of ductal carcinoma in situ diagnosed, with 44,000 deaths predicted in 2021 [1]. Furthermore, the reach of this disease is vast; 1 in 8 women will be diagnosed with invasive breast cancer in their lifetime [2].

Breast cancers are characterized into major classifications based on signaling receptor expression profile: hormone receptor positive estrogen receptor and/or progesterone receptor (ER and/or PR), human growth factor receptor-2 (HER2) positive, or triple-negative breast cancer (ER/PR/HER2 receptor negative or TNBC) [3]. Within these classifications, breast cancer is heterogeneous, underlying the difficulties presented to current therapeutic strategies. The ER+ and/or PR+ class is further classified into luminal A and B subgroups, which have differing levels of hormone sensitivity, cell proliferation, and prognosis [4]. The HER2+ subtype is identified through amplification and overexpression of HER2/neu, and has a high probability of response to chemotherapy along with therapeutics that target HER2, including pertuzumab and trastuzumab [5]. For hormone receptor and HER2 negative (triple negative) breast cancer patients, adjuvant chemotherapy has historically been the predominant therapeutic approach. Recently, immunotherapies such as the anti-PD1 pembrolizumab have been approved for certain indications in the treatment of this highly aggressive subtype [6,7]. In 2018, the implementation of a PARP inhibitor, olaparib, became the first targeted therapeutic for patients with a BRCA1 mutation and metastatic triple negative breast cancer [8]. 

Though significant advancements in current treatment strategies have been achieved, disease recurrence and drug resistance remain significant challenges in the field due to a multitude of factors associated with tumor cell heterogeneity [9]. Thus, understanding the fundamental biological mechanisms that affect breast cancer hormone sensitivity, immune surveillance, cell proliferation, cellular differentiation/phenotypic shifts, modification of the extracellular environment, and angiogenesis/metabolic capacity are crucial to developing novel treatment strategies that decrease cellular resistance and disease recurrence. One fundamental mechanism to these essential cellular processes is transforming growth factor-beta (TGFβ) signal transduction [10]. 

Following the discovery of transforming growth factor (TGF) in normal tissue and serum in the 1980s, the actions of the TGF protein family of polypeptides (including TGFβ, activin, inhibin, anti-Mullerian hormone, bone morphogenetic proteins [BMPs], growth differentiation factors [GDFs], and nodals) were found to have heterogenous roles in several aspects of development, homeostasis, and cancer [11]. Notably, in mammary tissue, TGFβ functions as a potent proliferation inhibitor and apoptosis inducer in early stages [12], yet promotes cancer aggressiveness in advanced stages of disease [13,14]. This paradoxical dual effect of TGFβ on cancer development and progression supports the investigation of TGFβ canonical and non-canonical pathways to advance the field of breast cancer therapeutics. Our review will focus on the implications of TGFβ signaling and regulation of this pathway using modifications of cell cycle/proliferation checkpoint inhibitors known as cyclin-dependent kinase (CDK) inhibitors, used to treat hormone receptor and human epidermal growth factor receptor 2 (HER2) positive and negative advanced breast cancer.

## 2. TGFβ Signaling: Canonical/SMAD, Non-Canonical/Non-SMAD, and Cross-Talk Pathways

**Figure 1:**
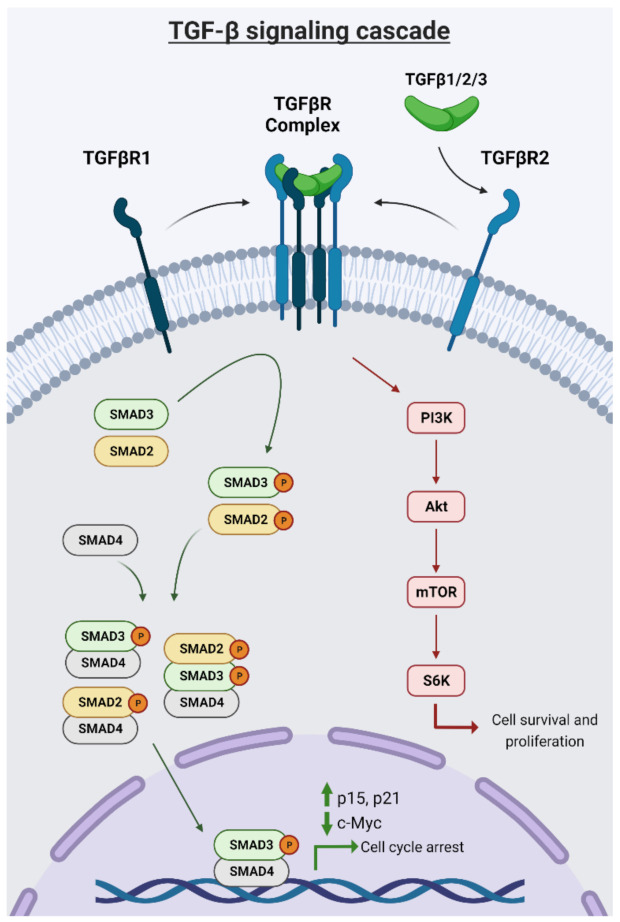
TGFβ signaling through canonical and noncanonical pathways. Image generated using Biorender.

TGFβ signaling proceeds through a complex cascade including multiple receptors, ligands, and transcriptional outputs (Figure 1). The TGFβ superfamily comprises a dimeric, disulfide-linked cytokine family of secreted ligand proteins, including three mammalian isotypes of TGFβ: TGFβ1, TGFβ2, TGFβ3, of which TGFβ1 is the most common [15]. TGFβ ligand binds with specific cell surface transmembrane receptors that have intrinsic serine/threonine kinase activity. TGFβs are initially secreted by cells and sequestered in an inactive form within the extracellular matrix, and these ligands are later activated in an integrin-dependent manner [16]. Activated TGFβs bind to TGFβ type II receptors (TβIIR), which recruit and phosphorylate TGFβ type I receptors (TβRI) at specific serine and threonine residues [17]. 

Subsequently, in canonical pathway signaling, TβRI phosphorylates SMAD2 and SMAD3 at C-terminal serine residues, which then assemble into heterodimeric and trimeric complexes with SMAD4 [17]. The SMAD complexes translocate to the nucleus to regulate several different TGFβ target genes, including c-Myc, p21, p15, Snail, Zeb1, Twist1, FoxH1, Mixer, Runx-related proteins, and E2F [18]. SMAD 3/4 bind directly to DNA after nuclear translocation; however, due to their weak affinity, SMADs rely on cooperation with other DNA-binding transcription factors, such as ATF2, Sp1, and Jun, for regulation of gene expression [19]. In the non-canonical setting, TGFβ signaling can be initiated through TβRII/TβRI receptors but then perpetuated through non-SMAD cascade patterns involving other protein kinases including PI3K, SHC/GRB2/SOS, TRAF4/6, and RHO/ROCK [20]. For example, activated TβRI can directly phosphorylate PI3K, which then phosphorylates AKT, activating mTOR, S6K and downstream effector genes of cell survival.

Beyond the canonical and non-canonical pathways downstream of TβRII/TβRI activation, TGFβ family protein cross-talk also acts to induce a myriad of growth regulatory effects. As TGFβ is part of a larger family of proteins that are structurally and functionally relatively similar (i.e., activins and BMPs), there is a vast repertoire of SMAD-interacting proteins and nuclear effector proteins, including Wnt, Hedgehog, Hippo, cytokine/JAK, and growth factor receptor/tyrosine kinase pathways [21]. Outcomes of these cross-talk interactions hinge largely on the milieu and context of localized cellular and environmental cues. As such, this work is specifically focused on the effects of TGFβ isoform (TGFβ1, β2, β3) signaling in the mammary environment and breast cancer. Interestingly, in breast oncogenesis, TGFβ1 and TGFβ2 overexpression has been linked with poor clinical outcomes, whereas TGFβ3 expression has been associated with a protective function [22]. 

## 3. Effects of TGFβ Signaling on Cell Cycle

Phosphorylation of SMAD2 and SMAD3 at the C-terminus by TβR1 prevents the G1-S phase transition of the cell cycle in healthy cells [23]. Initially, normal G1-S phase transition occurs through upregulation of cyclin D, the substrate of CDK4/6, which leads to phosphorylation of Rb and activation of E2F transcription factors. Subsequent upregulation of cyclin E, the substrate of CDK2, leads to the G1-S transition [24]. Canonically phosphorylated SMAD3 at the S423/425 site leads to downregulation of c-Myc and upregulation of CDK inhibitors p15 and p21 [25,26,27]. P15 inhibits CDK4/6 and p21 inhibits both CDK2 and CDK4 [25,26,27]. Upregulation of SMAD activity therefore leads to downstream cell cycle arrest, namely decreased Rb phosphorylation and decreased E2F activity leading to cell cycle repression.

Alternative phosphorylation sites within the SMAD proteins also play a crucial role in cell cycle regulation. SMAD3 is a physiological substrate of CDK2 and CDK4 at the T8, T178, S203, S207, and S212 sites [28]. One of these sites, T8, resides in the DNA binding region MH1 of the SMAD protein, while the other phosphorylation sites are located in the linker region of the protein between MH1 and MH2 [29]. Noncanonical phosphorylation can lead to degradation of the SMAD protein through ubiquitin ligase Nedd4L and thus, in certain situations, limits the effectiveness of TGFβ-mediated cell cycle arrest [30]. Additional mediators for noncanonical SMAD action include JNK, ERK1/2, p38, and GSK3b, all of which directly or indirectly influence the cell cycle [31,32,33]. Oncogenic upregulation of cyclins can disrupt the balance of activating and repressing factors, and lead to changes in signaling that affect cell cycle progression, dependent on the mechanism and differentiated state of the cancer.

## 4. Cell Cycle Dysregulation in Breast Cancer

Improvements in genomics and proteomic-based technology starting in the 1990s have enabled the investigation of cell cycle component behaviors in breast cancer tissue. As breast cancer biopsies were sequenced, it became apparent that a significant proportion of patient samples exhibited dysregulation of the CDK4/cyclin D1/Rb interaction with overexpression/amplification of cyclin D1 (CCND1) and alterations in p16 [34,35]. These sequencing data became more robust in the 2000s with the availability of the Cancer Genome Atlas, which elucidated that aberrations leading to hyperactivation of CCND1/CDK4/6 were specifically common in ER+ breast cancer subtypes [36]. Unsurprisingly, ER+ breast cancer that is resistant to endocrine therapy is often associated with CCND1 overexpression and Rb phosphorylation [37]. Importantly, the relationship between estrogen signaling and CDKs is bidirectional; CCND1 can independently activate the estrogen receptor in the absence of the endocrine signal [38]. Collectively, a substantial amount of evidence points toward a strategy targeting the TGFβ/SMAD/CDK pathway, which could have significant clinical and therapeutic implications for the treatment of breast cancer.

## 5. CDK Inhibitor Therapy in Hormone Receptor Positive (HR+) Breast Cancer

CDK inhibitors, either pan-CDK inhibitors or those targeting specific CDKs, have been the subject of numerous clinical trials, with varying efficacies (reviewed in [39]). To date, CDK inhibitor therapy, specifically inhibitors targeting CDK4 and CDK6, has found the most success in the treatment of HR+ breast cancer. Three such drugs—palbociclib, ribociclib, and abemaciclib—have been FDA-approved for the treatment of breast cancer. The PALOMA-2 [40] and PALOMA-3 [41,42] trials examined the CDK4/6 inhibitor, palbociclib, in combination with an aromatase inhibitor (letrozole, PALOMA-2) or an estrogen receptor degrader (fulvestrant, PALOMA-3). PALOMA-2 examined 666 postmenopausal patients with ER+, HER2^−^ disease that had not been previously treated for advanced disease. This study showed progression free survival in the combination palbociclib-letrozole therapy group at 24.8 months, compared with 14.5 months for the letrozole alone group. PALOMA-3 examined 521 patients with metastatic hormone receptor positive (ER+ or PR+, collectively HR+), HER2^−^ breast cancer that had disease progression after hormonal therapy (*p* < 0.00001). Similar to PALOMA-2, this study found an increase in progression free survival for patients treated with the combination palbociclib-fulvestrant compared to placebo-fulvestrant: 9.5 months at median for palbociclib-fulvestrant and 4.6 months at median for placebo-fulvestrant. An increase in overall survival from 29.7 months to 39.7 months was observed for patients that had previously responded to endocrine therapy but had subsequently relapsed (*p* < 0.0001). This study did not find a correlation between PIK3CA mutation or hormone receptor expression level and response to palbociclib. In both of these studies, neutropenia, leukopenia, anemia, and fatigue were the most commonly reported Grade 3 adverse events.

The MONALEESA-2 [43], MONALEESA-3 [44], and MONALEESA-7 [45] studies examined a separate CDK4/6 inhibitor, ribociclib, in similar contexts to palbociclib examined in the PALOMA studies. MONALEESA-2 examined the efficacy of the ribociclib-letrozole combination therapy in 668 postmenopausal women with HR+, HER2^−^ recurrent or metastatic breast cancer that had not previously received treatment for advanced disease. Combination therapy was observed to increase 18-month progression free survival rate from 42.2% in the placebo-letrozole group to 63.0% in the ribociclib-letrozole group (*p* < 0.001). MONALEESA-3 examined the combination of ribociclib with fulvestrant in as study of 484 HR+, HER2^−^ patients that had received up to one line of antiestrogen therapy for advanced breast cancer [44]. In this study, progression free survival was increased in the ribociclib group to 20.5 months compared to 12.8 months in the placebo-fulvestrant group (*p* < 0.001). Overall survival at 42 months was observed to be 57.8% for the ribociclib group and 45.9% for the placebo group [46]. Positive results were also observed in the MONALEESA-7 trial of ribociclib-letrozole treatment in 672 premenopausal women with advanced HR+, HER2^−^ breast cancer. Median progression free survival of 23.8 months with ribociclib-letrozole was observed, compared with 13.0 months for patients treated with letrozole alone (*p* < 0.0001). Similar results were observed for overall survival endpoints in this study [47]. At 42 months, the ribociclib-treated group was observed to have 70.2% overall survival, while the placebo group showed 46.0% overall survival. The percentage of patients who received antineoplastic therapy during the study period was the statistically equivalent (approximately 70% for each group). Similarly to the studies with palbociclib, neutropenia and leukopenia were the most commonly reported Grade 3 adverse events.

The MONARCH-2 [48] and MONARCH-3 [49] studies examined a third CDK4/6 inhibitor, abemaciclib, in the context of HR+, HER2^−^ breast cancer. Abemaciclib plus fulvestrant showed an increase in progression free survival in a study of 669 women with HR+, HER2^−^ breast cancer that had progressed while receiving neoadjuvant or adjuvant endocrine therapy. Progression free survival increased from 9.3 months to 16.4 months when compared to fulvestrant alone (*p* < 0.001), and overall survival increased by 9.4 months [50]. In combination with a nonsteroidal aromatase inhibitor, abemaciclib was found to significantly increase progression free survival for patients with advanced HR+, HER2^−^ breast cancer, relative to treatment with the aromatase inhibitor alone [49]. Overall, these three sets of trials showed similar results and safety profiles and demonstrated the utility of CDK 4/6 inhibitor therapy in the treatment of advanced HR+ breast cancers, particularly when combined with standard treatments.

Uncontrolled cell cycle progression driven by hormone receptor signaling has overlap with TGFβ signaling, and thus implications for CDK inhibitor therapy. Estrogen receptor signaling, the target of letrozole and fulvestrant, can lead directly to transcription of CCND1, which in turn leads to enhanced CDK4/6 activity and activation of the cell cycle [51]. Estrogen receptor signaling also indirectly promotes entry into the cell cycle and oncogenesis through a number of different pathways, including ERK1/2, AKT/PI3K, and JAK/STAT signaling [52]. Each of these mechanisms intersects with TGFβ signaling. Directly, estrogen receptor activation was shown to contribute to the inhibition of SMAD3 activation by TGFβ [53,54]. ERα has been demonstrated to physically interact with SMAD2 and SMAD3 upon activation, limiting phosphorylation of SMAD3, and therefore diminishing transcription of CDK inhibitors p15 and p21 [54]. ERα activation also limits the CDK-inhibiting effects of TGFβ signaling by promoting ubiquitination and degradation of SMAD2 and SMAD3 [55]. Conversely, TGFβ induced activation of SMAD4 induces apoptosis in cell lines expressing a high level of ERα, an effect that requires ERα expression [56].

## 6. CDK Inhibitors to Treat HER2+ Breast Cancer

CDK 4/6 inhibitors that have been FDA-approved for the treatment of HR+, HER2^−^ breast cancer (palbociclib, abemaciclib, ribociclib) are also being examined for their efficacy in the treatment of HER2+ disease, with numerous ongoing clinical trials. One such example for patients with HER2+, HR+ breast cancer, the monarchHER trial, was a phase 2 clinical trial examining the efficacy of abemaciclib with either the humanized HER2 antibody trastuzumab alone, or a combination of trastuzumab and fulvestrant, compared with standard-of-care treatment. This trial found the combination of abemaciclib, trastuzumab, and fulvestrant significantly increased progression free survival (8.3 months compared with 5.7 months), indicating that combining therapy targeting HER2 and HR signaling with a CDK4/6 inhibitor in HER2+, HR+ disease may be effective [57].

Amplified HER2 signaling results in oncogenesis through several different cell proliferative actions, including activation of the MAPK and AKT pathways, leading to stimulation of the cell cycle and uncontrolled cell division, similarly to HR+ disease. HER2 signaling also directly inhibits canonical TGFβ signaling through phosphorylation of SMAD3 at the S208 site, leading to a loss of the tumor-suppressor function of SMAD3 [58]. CCNE1 upregulation has been shown to confer resistance to trastuzumab [59]. Sensitivity to trastuzumab in CCNE1-high, HER2+ tumors can be restored through inhibition of CDK2 activity, either through treatment with a CDK2 inhibitor or through restoration of SMAD3 signaling, indicating that CDK inhibition may be a viable treatment modality against HER2+ tumors with altered TGFβ signaling [59,60]. 

## 7. CDK Inhibitors for the Treatment of Triple Negative Breast Cancer

Triple negative breast cancer (tumors that lack amplification of ER, PR, and HER2) remains difficult to treat and has a worse prognosis when compared to other breast cancer subtypes [61]. Triple negative breast cancer (TNBC) is often characterized by loss of RB1 function, amplification of CCNE1, and upregulation of p15, all of which are associated with TGFβ signaling as well as CDK inhibitor therapy [62]. TNBC cell lines show sensitivity to CDK inhibition in preclinical models with upregulated CCNE [63,64,65], and clinical trials are working towards translating this result into clinical practice [66]. 

One of the main oncogenic results of upregulated CCNE in TNBC is the loss of the tumor suppressor function of TGFβ through increased noncanonical phosphorylation of SMAD3 [67]. Loss of canonical SMAD3 function can lead to downregulation of the CDK inhibitors p15 and p21 and upregulation of c-Myc, resulting in uncontrolled proliferation. MYC activation has been shown to be a synthetic lethal when combined with CDK inhibition in TNBC, thus solidifying the connection between CDK inhibitor therapy and TGFβ/SMAD3 signaling [68]. Prior work has extensively examined the effects of CDK/cyclin-mediated phosphorylation of SMAD3 in TNBC and the concomitant effects of CDK inhibitor therapy in this cancer subtype [63,64,65,69]. After treatment with a CDK2 or CDK4 inhibitor, TNBC cell lines that overexpress CCNE1 have increased canonical SMAD3 activity and decreased invasiveness and tumor growth [65]. A similar result was achieved by restoring canonical SMAD3 function through expression of a SMAD3 construct with mutated noncanonical CDK phosphorylation sites. Inhibition of CDK-mediated SMAD3 phosphorylation through the use of a CDK2/9 inhibitor additionally disrupted the Pin1-SMAD3 interaction which led directly to decreased aggressiveness in TNBC cells [69]. Pin1 is a cis-trans isomerase that promotes TGFβ induced migration [70] and association of Pin1 with SMAD3 promotes SMAD3 degradation [71], leading to down-regulation of the CDK-inhibition effects of TGFβ. A CDK2 inhibitor was additionally shown to have a synergistic effect with eribulin and paclitaxel in preclinical models of TNBC [71]. In these studies, CDK2 inhibition limited TNBC cell colony formation, migration, and tumor growth, and had increased expression of SMAD3 targets p15 and c-Myc, the effects of which were augmented through the addition of chemotherapy. This work further supports the hypothesis that the combination of CDK2 therapy with chemotherapy may be an effective treatment strategy for patients with TNBC. 

## 8. TGFβ-Mediated Resistance to CDK Inhibitors

CCND1 expression and subsequent CDK4/6 activity have been shown to promote breast oncogenesis [72]. An ever-growing body of clinical evidence has elucidated potential mechanisms of resistance to CDK inhibition, some of which are linked to TGFβ signaling [73]. Loss of RB function can render tumors unresponsive to the CCND1/CDK4/6 checkpoint in the cell cycle and thus limit the efficacy of CDK4/6 inhibitors [74,75], a finding that has been correlated with clinical response data through an analysis of and RB signature on the publicly available METABRIC dataset and other published data [76]. Overactivity of the CCNE1/CDK2 axis has also been shown to confer resistance to CDK4/6 inhibitors [77,78], and CCNE1/CDK2-mediated resistance to CDK4/6 therapy can potentially be overcome through the addition of a CDK2 inhibitor to the treatment [79]. TGFβ signaling can limit activation of the CCNE/CDK2 axis through upregulation of SMAD3 and transcription of the CDK2 inhibitor p21 [80]. Conversely, upregulated CCNE1 can lead to dysregulated TGFβ signaling through CDK2-mediated phosphorylation and subsequent degradation of SMAD3 [60]. Therefore, TGFβ signaling may be protective against CCNE-mediated resistance to CDK4/6 inhibitors through upregulation of p21 and subsequent inhibition of CCNE1/CDK activity.

Signaling through TGFβ can influence therapeutic outcomes in HER2+ breast cancer, which may have an effect on CDK inhibitor therapy. Filipits et al. used data from the TransHERA study to examine the prognostic value of CCND and expression of the CDK inhibitor p27 for the treatment of HER2+ breast cancer [81]. This study observed a significant correlation between p27 expression and efficacy of trastuzumab treatment when comparing patients in the treatment and placebo arm of the study. The study suggested that low p27 was necessary for efficacy of trastuzumab. Since p27 is a CCNE/CDK2 inhibitor, it is possible that a high level of CCNE/CDK2 inhibition through TGFβ signaling (via SMAD3-mediated p21 upregulation) could confer resistance to trastuzumab, and introduction of a CDK2 inhibitor could counteract that effect. Constitutive activation of TGFβR1 through the T204D mutation has been observed to result in resistance to trastuzumab and poor prognosis in HER2+ breast cancer patients [82]. CCNE amplification has been shown to correlate with poor clinical outcomes in patients with HER2+ breast cancer [83] and to result in resistance to trastuzumab in preclinical models [59]. This resistance to trastuzumab may be mediated by noncanonical phosphorylation of SMAD3 [60]. CCNE1 upregulation lead to resistance to trastuzumab and accompanying downregulation of SMAD3. Downregulation of SMAD3 was mediated by degradation due to increased phosphorylation at the T179, S204, and S213 linker region sites. Overexpression of a mutant SMAD3 construct with inhibited phosphorylation at the T179, S204, and S213 sites [28] restored the CDK-inhibitory effects of SMAD3 signaling and resensitized CCNE-high, HER2+ tumors to trastuzumab [60]. Importantly, CCNE-high, HER2+ tumors were sensitive to CDK2/9 inhibition [59,60], supporting the potential for CDK inhibitor therapy to serve as promising modality for the treatment of trastuzumab resistant HER2+ breast cancer.

## 9. Future Directions: TGFβ and CDK Inhibitors in the Breast Cancer Microenvironment

**Figure 2:**
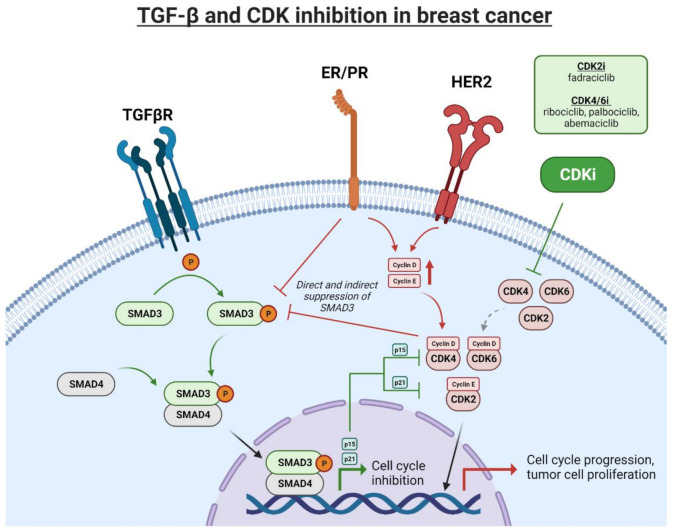
Interconnected signaling pathways between oncogenic activation of CDKs and TGFβ. Image generated using Biorender.

TGFβ signaling has the canonical effect of behaving as a tumor suppressor when acting directly on cancer cells through canonical SMAD signaling and subsequent upregulation of native CDK inhibitors (Figure 2). These functions are lost when counterbalancing signaling molecules are upregulated (e.g., ER, HER2, CCNE), a function which can be restored using CDK inhibitor therapy [60,63,65,69]. However, TGFβ also plays a suppressive role in the tumor microenvironment, which can limit the efficacy of cytotoxic therapies [84,85,86]. TGFβ signaling promotes a cancer-associated fibroblast phenotype [87], which can lead to epithelial-mesenchymal transformation in cancer cells [88] and immunosuppression more generally within the tumor [89]. TGFβ signaling impacts tumor infiltrating immune cells as well. Macrophages activated by TGFβ trend towards an anti-inflammatory phenotype that can promote tumor progression [90]. Importantly, these cells also produce TGFβ, which, when signaling in tumor cells that have lost the canonical suppressive function of TGFβ, can lead to increased invasiveness and dissemination of cancer [91,92]. TGFβ signaling in CD4+ T cells promotes a regulatory T cell phenotype [93], which has been associated with immunosuppression and poor prognosis in breast cancer [94,95,96,97]. In terms of cytotoxic T cells, TGFβ signaling can lead to anergy through upregulation of PD1-PDL1 signaling [98], thus limiting the efficacy of T cell-based therapies.

**Figure 3:**
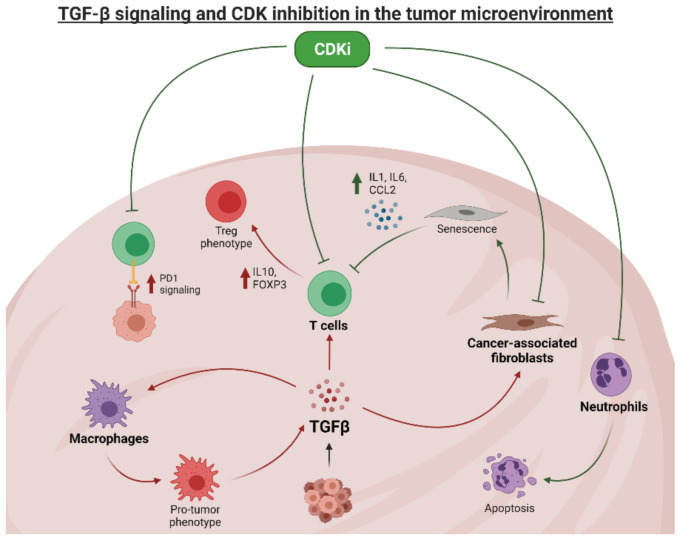
TGFβ signaling and CDK inhibition affect the breast cancer microenvironment. Image generated using Biorender.

CDK inhibitor therapy also has the effect of reversing some of the tumorigenic aspects of the breast cancer microenvironment (Figure 3). One of the most common side effects of CDK inhibitor therapy is severe neutropenia [41,44,45,48,49,50]. Numerous preclinical models of breast cancer have implicated neutrophils in metastatic progression [63,99,100,101,102,103,104], and depletion of suppressive neutrophils has been suggested as a possible therapeutic modality for breast cancer [105]. Fibroblasts that are forced into the G1 phase, either through advanced aging or the use of a CDK inhibitor, adopt a senescent phenotype [106]. These senescent fibroblasts secrete IL1, IL6, and CCL2 [107], all of which polarize cytotoxic lymphocytes towards an antitumor phenotype. As such, CDK inhibitors may be a potential means for overcoming CAF-mediated immunosuppression.

Mounting evidence suggests that CDK inhibitor therapy can augment antitumor immune responses and potentially overcome the immunosuppressive aspects of TGFβ signaling. Data has shown that inhibition of TGFβ signaling can enhance the efficacy of checkpoint blockade therapy, an effect that can be achieved through CDK inhibitor therapy [108,109,110]. Indeed, treatment with a CDK inhibitor appears to synergize well with checkpoint blockade therapy, as CDK4/6 inhibitors have been shown to significantly enhance T cell activation and augment the response to PD1 blockade in preclinical models of breast and lung cancers [111,112]. Mechanistically, CCND-CDK4 action was shown to regulate PD-L1 expression through phosphorylation of speckle type BTB/POZ protein (SPOP), leading to degradation of SPOP and subsequent loss of ubiquitination-mediated PD-L1 degradation [113]. Preliminary reports suggest that these findings may extend to the clinical treatment of breast cancer. The neoMONARCH trial showed neoadjuvant abemaciclib in combination with anastrozole enhanced antigen presentation and activated T-cell phenotypes in HR^+^/HER2^−^ breast cancer patients [114]. Together, these results suggest that adjuvant CDK inhibitor therapy may also enhance the efficacy of checkpoint blockade in tumors that overexpress PD-L1. These results are particularly relevant to the treatment of TNBC. In the recently reported KEYNOTE-522 trial, pembrolizumab (anti-PD-1) treatment along with chemotherapy, implemented for the treatment of stage II or stage III TNBC, was shown to significantly increase the percentage of patients who experienced a pathological complete response to neoadjuvant therapy (64.8% in 782 patients, compared with 51.2% in 390 patients treated with placebo-chemotherapy, *p* < 0.001) [6]. An exciting future therapeutic strategy in preclinical investigation includes a combination of CDK inhibition with anti-PD-1 therapy, which may enhance these trial results and provide additional benefit to patients with high-risk TNBC. 

## 10. Conclusions

CDK inhibitor therapy holds great promise for the treatment of breast cancer. TGFβ/SMAD3 signaling can significantly impact the outcome of CDK inhibitor therapy, both through canonical signaling as a cell-cycle inhibitory pathway, as well as through immunosuppressive effects in the cancer microenvironment. Active initiatives to further the understanding of the role that aberrant TGFβ/SMAD3 signaling plays in the sensitivity to CDK inhibitor therapy will lead to more precise implementation of this cancer treatment modality for the management of breast and other cancers in the near future.

## Data Availability

The data presented in this study are available within the article.
